# Protein kinase A inhibits tumor mutator APOBEC3B through phosphorylation

**DOI:** 10.1038/s41598-019-44407-9

**Published:** 2019-06-05

**Authors:** Tadahiko Matsumoto, Kotaro Shirakawa, Masaru Yokoyama, Hirofumi Fukuda, Anamaria Daniela Sarca, Sukenao Koyabu, Hiroyuki Yamazaki, Yasuhiro Kazuma, Hiroyuki Matsui, Wataru Maruyama, Kayoko Nagata, Fumiko Tanabe, Masayuki Kobayashi, Keisuke Shindo, Ryo Morishita, Hironori Sato, Akifumi Takaori-Kondo

**Affiliations:** 1 Department of Hematology and Oncology, Graduate School of Medicine, Kyoto University, Kyoto, 606-8507 Japan; 2 Laboratory of Viral Genomics, Pathogen Genomics Center, National Institute of Infectious Diseases, Tokyo, 208-0011 Japan; 3 CellFree Sciences Co., Ltd, Ehime, 790-8577 Japan

**Keywords:** Oncogenes, Phosphorylation

## Abstract

APOBEC3B cytidine deaminase (A3B) catalyzes cytosine into uracil in single-strand DNA and induces C-to-T mutations in genomic DNA of various types of tumors. Accumulation of APOBEC signature mutations is correlated with a worse prognosis for patients with breast cancer or multiple myeloma, suggesting that A3B activity might be a cause of the unfavorable DNA mutations and clonal evolution in these tumors. Phosphorylation of conserved threonine residues of other cytidine deaminases, activation induced deaminase (AID) and APOBEC3G, inhibits their activity. Here we show that protein kinase A (PKA) physically binds to A3B and phosphorylates Thr214. *In vitro* deaminase assays and foreign DNA editing assays in cells confirm that phosphomimetic A3B mutants, T214D and T214E, completely lose deaminase activity. Molecular dynamics simulation of A3B phosphorylation reveals that Thr214 phosphorylation disrupts binding between the phospho-A3B catalytic core and ssDNA. These mutants still inhibit retroviral infectivity at least partially, and also retain full anti-retrotransposition activity. These results imply that PKA-mediated phosphorylation inhibits A3B mutagenic activity without destructing its innate immune functions. Therefore, PKA activation could reduce further accumulation of mutations in A3B overexpressing tumors.

## Introduction

The human apolipoprotein B mRNA editing enzyme, catalytic subunit (APOBEC) protein family comprises 11 DNA and RNA deaminases: activation induced cytidine deaminase (AID), APOBEC1, APOBEC2, APOBEC3A to H and APOBEC4^1^. APOBEC deaminases play pivotal roles in multiple cellular phenomena including cancinogenesis, metabolism, and innate and adaptive immunity^2,3^. APOBEC proteins deaminate cytosine into uracil, resulting in cytosine to thymine mutations in human and viral genomes. AID, A1, A3A, A3C and A3H are single cytidine deaminase domain (CDD) proteins with one zinc-coordinating active site, while A3B, A3D, A3F, and A3G are double CDD proteins with two zinc-coordinating motifs. AID plays a crucial role in somatic hypermutation and class switch recombination, which enables diversification of immunoglobulin genes in germinal center B cells^4^. Another APOBEC protein, APOBEC3G (A3G), is a potent anti-retroviral host factor against HIV-1^5–8^ and HTLV-1^9^. A3G is incorporated into budding virions and induces C-to-T transitions in the minus-strand of the viral DNA, inhibiting HIV-1 replication.

One of the APOBEC deaminases, APOBEC3B (A3B) has been recently recognized as an intrinsic DNA mutator in various types of cancer. Whole genome sequencing demonstrated that A3B contributes to mutation, clonal evolution, chemotherapy resistance and poor outcome of several types of cancers, including breast^10–15^, lung^11,16,17^, urothelial^11,16,18^, head and neck^11,16^ and serous ovarian^19^ cancers. We previously showed that A3B induces APOBEC signature mutations in genomic DNA in a cell based model^20^. Accumulation of APOBEC signature mutations correlates with a worse prognosis for patients with breast cancer^15^ or multiple myeloma^21–23^, suggesting that APOBEC induced mutations could affect clonal evolution or genomic instability of cancers, in other words, lead to resistance to chemotherapy and malignant transformation. The data so far highlights that inhibition of A3B activity could be an attractive therapeutic strategy in A3B expressing cancers.

Transcriptional regulation of A3B expression has been extensively investigated by several groups, including our own. In several types of cancers, canonical^24^ or non-canonical NF-κB^25^, b-Myb^26^ and human polyomavirus infection^27,28^ induce A3B transcription. On the other hand, post-translational regulation of A3B is not yet fully elucidated. A simian viral protein, SIV-Vif, was reported to form an E3 ubiquitin ligase complex with cellular proteins, poly-ubiquitinate and degrade A3B^29^, but, so far, there have been no reports describing post-translational modifications of A3B by intrinsic cellular factors.

Post-translational regulation of APOBEC proteins has received considerable attention after APOBEC3G (A3G) was originally identified as a target of the viral E3 ubiquitin ligase complex^30–32^. HIV-Vif forms an ubiquitin ligase complex with cellular proteins Cul5, EloB/C, Rbx1, which binds and degrades A3G in order to promote HIV infection. Protein kinase A (PKA)-mediated phosphorylation is a common regulatory mechanism for AID and A3G functions. PKA phosphorylates AID at Thr27 and Ser38, the latter necessary for AID and RPA2 binding, which promotes class switch recombination^33–35^. PKA also phosphorylates A3G both at Thr32 and at Thr218. Phosphorylation of Thr32 reduces the affinity between A3G and HIV Vif, and renders the former less susceptible to Vif-induced degradation^36^. Phosphorylation of both A3G Thr218 and AID Thr27 suppresses their cytidine deaminase (CDA) activity^35^. PKA-mediated phosphorylation may be a common molecular switch among some of the APOBEC family members.

PKA is a prominent serine/threonine kinase, also called cAMP-dependent protein kinase^37^. In an unstimulated state, PKA resides in the cytoplasm as an inactive heterotetramer holoenzyme, comprised of two catalytic subunits and two regulatory subunits. Upon stimuli that elevate the concentration of cAMP in the cytoplasm, two cAMP molecules bind to the regulatory subunits and induce a conformational change, which allows the catalytic subunits to dissociate and phosphorylate downstream targets, not only in the cytoplasm but also in the nucleus^38,39^. The most common motif in the physiological substrates of PKA is R(R/X)X(S/T)^40^. We hypothesized that A3B is also a target of PKA and that PKA-mediated phosphorylation serves as an A3B activity control mechanism. In this study, we show that PKA physically binds to and phosphorylates Thr214 of A3B and that PKA-mediated phosphorylation of this residue inhibits its deaminase activity.

## Results

### A3B Ser46 and Thr214 are putative PKA phosphorylation sites

**Figure 1:**
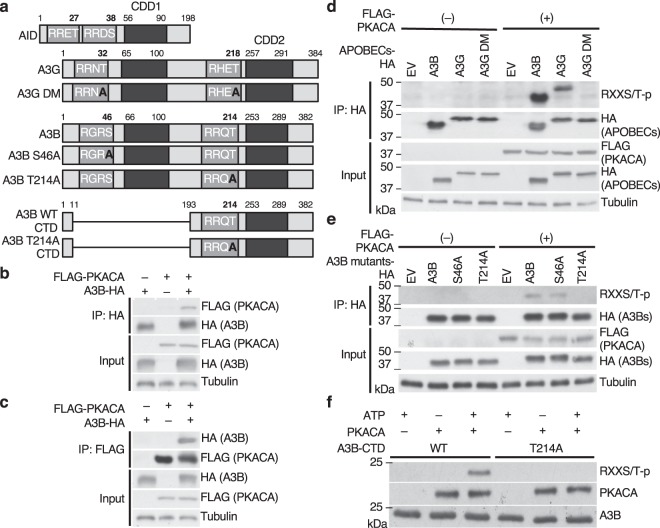
PKACA phosphorylates A3B Thr214 *in vivo* and *in vitro*. (**a**) Schematic figure of AID, A3G, A3B and their mutants. A3B WT CTD and A3B T214A CTD are the purified protein constructs. (**b,c**) A3B physically binds to PKACA. HEK293T cells were transfected with expression vectors for A3B-HA and FLAG-PKACA. Lysates from these cells were co-immunoprecipitated with anti-HA (**b**) or anti-FLAG antibodies (**c**), followed by immunoblot with the indicated antibodies. (**d**) PKACA phosphorylates A3B in HEK293T cells. HEK293T cells were transfected with expression vectors for A3B, A3G, and A3G DM with or without PKACA, as shown above. 36hrs after transfection, we semi-purified the APOBECs by immunoprecipitation, and PKA-mediated phosphorylation was detected by immunoblot with anti-RXXS/T-p antibodies. (**e,f**) PKACA phosphorylates Thr214 of A3B. **(e)** Expression vectors for A3B alanine mutants with or without PKACA are introduced in HEK293T cells as indicated. 36 hrs after transfection, cells were collected, lysed, immunoprecipitated with the anti-HA antibody and subjected to immunoblot with the indicated antibodies. The results show that A3B Thr214 is a crucial residue for PKA-mediated phosphorylation. (**f**) *In vitro* phosphorylation assays show that PKACA directly phosphorylates A3B-CTD, but not A3B T214A CTD. AID, activation-induced deaminase; WT, wild type; DM, double mutant; IP, immunoprecipitation.

Previous studies reported that PKA phosphorylates Thr32^32^, Thr218^35^ of A3G and Thr27^33^ of AID, which changes their enzymatic activity or physiological function. We identified Ser46 and Thr214 of A3B within consensus PKA phosphorylation motifs homologous to AID and A3G (Supplementary Fig. 1). The two amino acids (Ser46 and Thr214) are located in the CDD of A3B, similar to known phosphorylation sites of AID and A3G (Fig. 1a). In addition, two phosphorylation prediction tools, NetPhosK1.0 (http://www.cbs.dtu.dk/services/NetPhosK/)^41^ and ScanSite (http://scansite.mit.edu)^42^, singled out Ser46 and Thr214 of A3B as putative PKA-mediated phosphorylation sites.

### PKA physically binds to A3B and directly phosphorylates Thr214

First, we demonstrated the physical binding between A3B and PKA catalytic subunit α (PKACA). We expressed C-terminal HA–tagged A3B (A3B-HA) and N-terminal FLAG-tagged PKACA (FLAG-PKACA) in HEK293T, followed by co-immunoprecipitation (Co-IP). FLAG-PKACA co-precipitated with A3B-HA (Fig. 1b). This binding was confirmed by reciprocal Co-IP assays (Fig. 1c). We then examined whether PKACA actually phosphorylates A3B in the transfected cells. After immunoprecipitating A3B-HA using anti-HA antibodies, we performed immunoblot analyses using anti-phospho-PKA substrate (anti-RXXS/T-p) antibodies and detected the phosphorylated A3B (Fig. 1d). We further showed that overexpressing the kinase-dead PKACA mutant, K72H^43^, did not induce A3B phosphorylation (Supplementary Fig. 2). These data indicate that PKACA binds to and phosphorylates A3B in HEK293T cells.

Next, to investigate which A3B residues are phosphorylated by PKA, we generated alanine substituted mutants of the predicted phosphorylated residues, S46A or T214A respectively (Fig. 1a). We transfected HEK293T cells with expression vectors for these mutants along with FLAG-PKACA. Immunoblotting with anti-RXXS/T-p antibodies revealed that PKACA phosphorylates wild type (WT) and S46A, but not T214A A3B-HA (Fig. 1e), suggesting that Thr214 is the PKA phosphorylation site of A3B. Co-IP assays revealed that the A3B T214A mutant still binds to PKACA, suggesting that the lack of phosphorylation of T214A mutant is not due to loss of direct interaction between T214A and PKA (Supplementary Fig. 3). We purified WT A3B C-terminal domain and its mutants (Supplementary Fig. 4) using wheat germ cell free expression system^44^ to perform *in vitro* phosphorylation assays. These assays confirmed that PKACA directly phosphorylates WT A3B-CTD, but not the T214A mutant (Fig. 1f).

### Phosphomimetic A3B Thr214 mutants lose their cytidine deaminase activity *in vitro*

**Figure 2:**
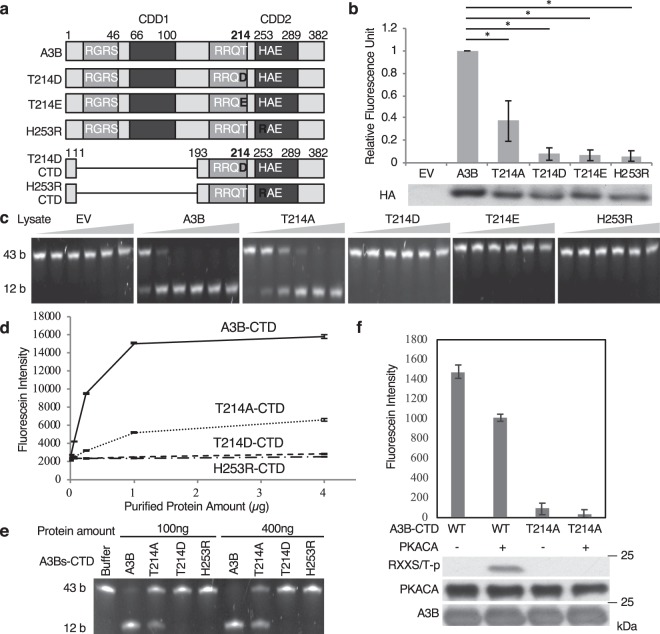
Phosphomimetic A3B mutants lose their CDA *in vitro*. (**a**) Schematic figure of phosphomimetic A3B mutants at Thr214. T214D CTD and H253R CTD are the purified protein constructs. (**b**) Phosphomimetic A3B mutants lose their CDA *in vitro*. FRET-based CDA assays were performed using lysates from HEK293T cells with exogenously-expressed A3B or its mutants. Each of the fluorescent intensity numerical values represent the average of three independent experiments, normalized to that of the WT sample. Bars indicate standard error (SE), and asterisks (*) show statistically significant difference (p < 0.05) (upper panel). The protein levels of the A3B mutants used in the experiment were comparable (lower panel). (**c**) Phosphomimetic A3B mutants lose CDA against the TCA sequence in the gel-based CDA assay. In this assay A3B CDA catalyzes substrate 43b oligo to 12b oligo transition. Lysates from cells transfected with EV, T214D, T214E or H253R have no CDA, and those with T214A have less CDA than those with WT. (**d**) FRET-based CDA assays using purified proteins. Purified C-terminal A3B T214D almost completely loses CDA *in vitro*. Each of the fluorescent intensity numerical values represent the average of three independent experiments. (**e**) Gel-based CDA assay using purified proteins. A3B T214D loses CDA, and T214A has weak CDA against the TCA sequence. (**f**) Phosphorylation of A3B reduces its cytidine deaminase activity. A3B-CTD and T214A-CTD were phosphorylated by PKACA *in vitro*, and subsequently performed *in vitro* CDA assays. Values represent the average of two independent experiments, and bars indicate SE.

According to the CDD C-terminal crystal structure, Thr214 participates in the formation of the catalytic pocket^45,46^ and seems to interact with substrate cytosine in single strand DNA (ssDNA) to hold it in an appropriate position^47^. We investigated whether PKA-mediated phosphorylation of this residue affects the molecular function of A3B, deamination of cytosine in 5′-TCX sequences of ssDNA. We performed fluorescence resonance energy transfer (FRET)-based CDA assays^48^, using cell lysates with overexpressed WT A3B or its mutants, T214A, T214D and T214E (phosphomimetic mutants), and H253R (CDA abrogated mutant) (Fig. 2a), and oligo ssDNA containing one target sequence, 5′-TC, with a FAM-TAMRA reporter-quencher pair at its ends. If the substrate oligonucleotide is intact, there is no fluorescence. When A3B catalyzes the deamination of cytosine to uracil, which is then excised and the oligonucleotide is cleaved at the remaining abasic site, the quenching effect is lost and fluorescence regained (Supplementary Fig. 5a–d)^48^. With these assays, we show that lysates containing the T214A mutant have about one-third of the WT A3B CDA activity, and the phosphomimetic mutants have little to no activity, comparable to that of the empty vector or H253R (Fig. 2b upper panel). The protein levels of A3B or its mutants in the lysates are comparable (Fig. 2b, lower panel).

To ascertain that the CDA activity reduction is TC sequence specific, we performed Gel-based CDA assays using serially diluted lysates. The initial 43-base long, 5′ FAM-labeled ssDNA (substrate) is subjected to A3B CDA activity. The ensuing cytosine to uracil transition, uracil excision and cleavage of the newly formed abasic site lead to the formation of a 12-base long, 5′ FAM-labeled oligo DNA (product) (Supplementary Fig. 5e–h). If the substrate is cleaved through a different mechanism, the product length will differ from 12 bases^48^. As expected, lysates containing A3B T214D or T214E lack TC sequence specific CDA activity, similar to that containing A3B H253R (Fig. 2c). The lysate of A3B T214A expressing cells preserves this function, albeit weaker than that of A3B WT.

To confirm the changes observed for each mutant, we conducted FRET-based CDA assays using purified A3B-CTD and its mutants. T214D and H253R have almost no CDA activity, and T214A has reduced activity compared to WT A3B-CTD (Fig. 2d). Using gel-based CDA assays, we confirmed that T214D and H253R lose TC sequence specific CDA activity, while T214A preserves only a fraction of the WT A3B activity (Fig. 2e).

We further investigated the CDA activity of *in vitro* phosphorylated A3B. We incubated purified A3B with purified PKACA protein and ATP, and measured the CDA activity of partially phosphorylated A3B using FRET-based *in vitro* CDA assays. The result showed that phosphorylated A3B has reduced CDA activity compared to WT sample (Fig. 2f). Taken together, phosphorylation of Thr214 inhibits A3B CDA activity *in vitro*.

### A3B phosphomimetic mutants preserve nuclear localization

The subcellular localization of A3B is well described^12,19,49^, and predominantly limited to the nucleus. To assess whether our A3B mutants retain normal, steady-state subcellular distribution, we carried out a series of live cell A3B localization studies. We transfected HeLa cells with expression vectors for WT or mutants of C-terminal FLAG-tagged A3B, followed by immunofluorescence staining with anti-FLAG antibodies. As previously reported, WT A3B localized exclusively in the nucleus, and the T214A, T214D, T214E, and H253R A3B mutants also concentrated in the nucleus (Supplementary Fig. 6). These data suggest that PKA mediated phosphorylation does not affect A3B subcellular localization.

### Molecular dynamics simulation reckoned that Thr214-phosphorylation and phosphomimetic Glu/Asp at 214 induce dissociation of cytosine from the catalytic pocket

**Figure 3:**
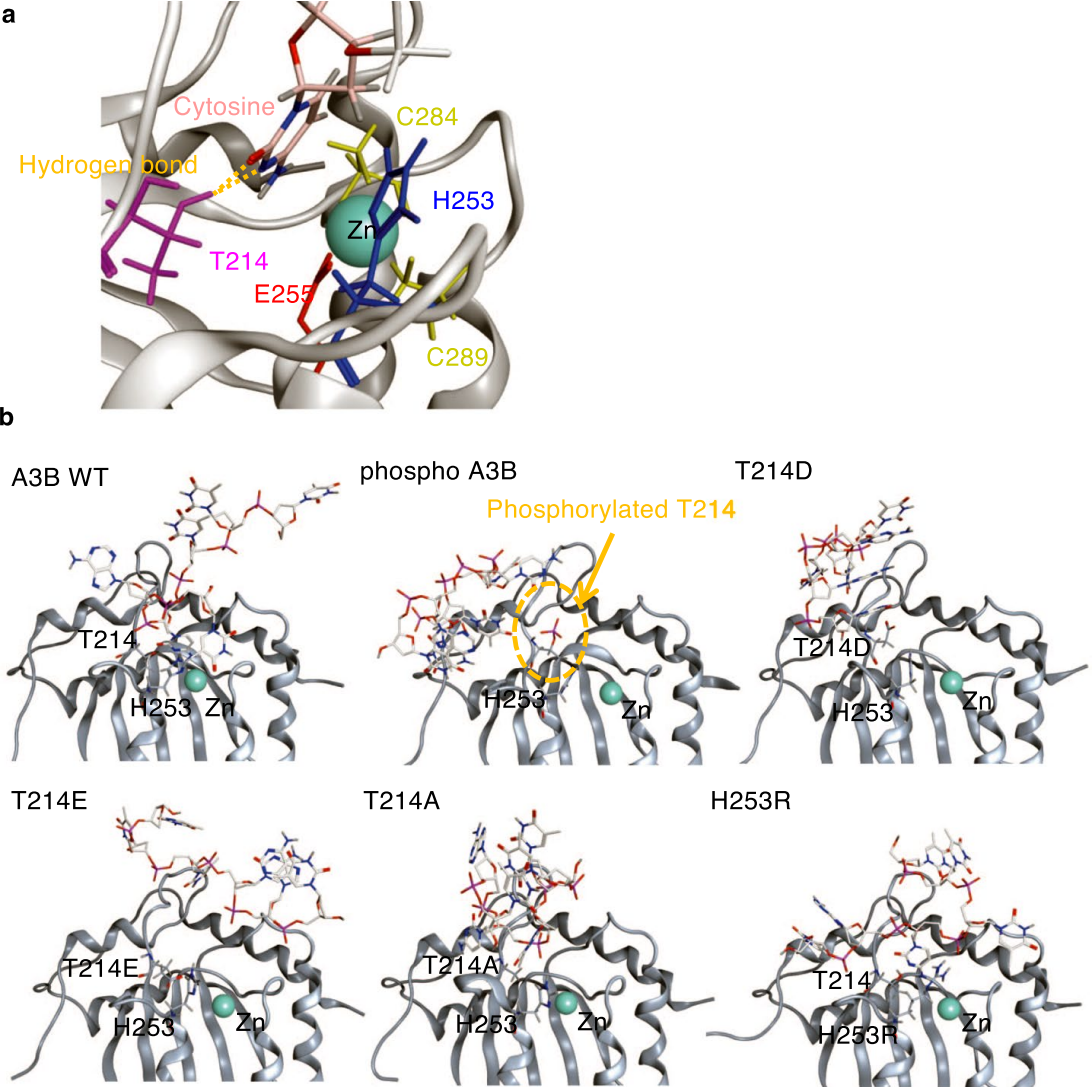
A3B phosphorylation or mutation affects ssDNA binding. Structural models for A3Bs in an ssDNA-bound state were constructed with reported X-ray crystal structures of A3B (PDB number 5CQH) and ssDNA (PDB number 5TD5), and subjected to MDS as described in the experimental procedures section. (**a**) A structure in an ssDNA-bound state at thermodynamic equilibrium, for 80 ns of MDS of the A3B WT-ssDNA complex model. The active site of the enzyme is highlighted. A3B residues (T214, H253, E255, C284, and C289), located near cytosine and Zn, and presumably involved in the chemical catalysis of DNA cytidine deamination, are shown in color sticks. Orange dotted lines indicate hydrogen bonds between Thr214 and cytosine. (**b**) Binding modes of ssDNA with various A3Bs (A3B WT, phosphorylated, T214D, T214E, T214A, and H253R) after 80 ns of MDS. A3B active centers around Zn and ssDNA are highlighted.

To examine how phosphorylation or various substitutions of A3B Thr214 influence A3B-ssDNA molecular interactions, we examined the A3B and ssDNA binding mode using molecular dynamics simulations (MDS). In the WT, non-phosphorylated A3B, Zn^2+^ was surrounded by His253, Glu255, Cys284, and Cys289 in the catalytic pocket located in the vicinity of a cytosine of the ssDNA which formed a hydrogen bond with the side chain of Thr214 (Fig. 3a,b A3B WT). When Thr214 was phosphorylated, the cytosine could not enter the pocket due to the repulsion between the negative charges of the phosphate group and ssDNA (Fig. 3b phospho A3B). Similar dissociation of the cytosine from the pocket was observed with the phosphomimetic A3B mutants, T214D and T214E (Fig. 3b T214D and T214E), and with the T214A mutant, the latter probably due to the lack of hydrogen bond formation with the side chain of alanine (Fig. 3b T214A). In contrast, the H253R mutant showed no significant changes in the relative positions of the cytosine, Zn^2+^, and Glu255 in the A3B pocket (Fig. 3b H253R). The results suggest that Thr214 also plays an important role in the nucleophilic reaction of A3B-induced cytosine deamination. These structural data are well consistent with the phenotypic changes of the A3B variants in the present study.

### Phosphomimetic A3B mutants lose foreign DNA editing activity

Cellular A3B is known to possess extrinsic DNA, or transfected plasmids, editing activity^20^. To explore whether A3B phosphorylation alters this function, we performed foreign DNA editing assays. DNA from HEK293T cells transfected with expression vectors for WT, T214A, T214D, T214E or H253R A3B-HA mutants along with pEF-UGI and pDON-EGFP as catalytic substrates, was used for differential DNA denaturation PCR (3D-PCR) assays^20^. 3D-PCR is used to detect the amount of C-to-T mutations, based on the rationale that nucleoside alterations from C-G to A-T pairs decrease the number of hydrogen bonds within double stranded DNA, lowering its denaturation temperature (Td). As a result, DNA containing C-to-T mutations can be amplified at a lower Td compared to mutation-free DNA.

**Figure 4:**
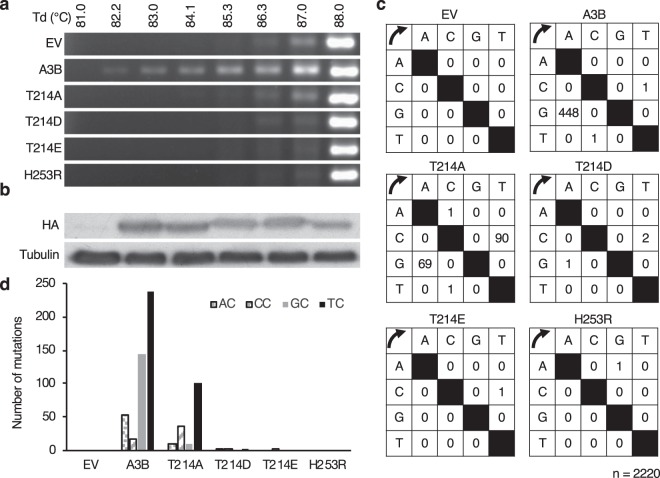
Phosphomimetic A3B mutants lose foreign DNA editing activity. (**a**) 3D-PCR of EGFP exposed to WT A3B and its mutants. HEK293T cells were co-transfected with expression vectors for A3B or its mutants together with pDON-EGFP and pEF-UGI. Total DNA was recovered 2 days after transfection, and the EGFP gene was amplified by 3D-PCR at the indicated denaturation temperatures (Td). (**b**) Expression levels of A3B WT and its mutants were comparable. (**c**) Mutational matrix of EGFP sequences derived from PCR products amplified at the lowest Td in each sample. (**d**) Dinucleotide context of foreign DNA editing. The rates of C-to-T transitions in the indicated dinucleotide sequence are shown.

Robust EGFP PCR products are obtained at a Td of 88 °C in all samples. In samples containing the EV or A3B-HA T214D, T214E, or H253R mutants, there is no relevant amplification at a Td under 88.0 °C. In those containing T214A, PCR products are not detected under 86.3 °C, and samples with WT A3B-HA displayed amplicons as low as 82.2 °C (Fig. 4a). These results suggest that phosphomimetic A3Bs have virtually no foreign DNA editing activity, same as the EV or H253R, and the T214A mutant has weaker activity than WT. In these assays, the protein levels of the A3B mutants are comparable (Fig. 4b).

To confirm the A3B mediated DNA editing, we sequenced the PCR products obtained at the lowest Td in each sample. EGFP sequences are highly edited in the WT A3B-HA sample, with numerous C/G to T/A mutations, less so in the T214A sample, while only with residual mutations in the T214D and T214E samples and seemingly no or sporadic mutations in the EV and H253R samples (Fig. 4c,d). A3B prefers to deaminate cytosine in a 5′-TC sequence, so we looked at the context of C/G to T/A transitions in the hyperedited EGFP sequences. We observed a strong bias towards deamination in 5′-TC dinucleotides in WT and T214A samples. These results coincide with the *in vitro* A3B molecular functional changes induced by PKA-mediated phosphorylation described above.

### Phosphomimetic A3B mutants retain anti-retroviral activity

**Figure 5:**
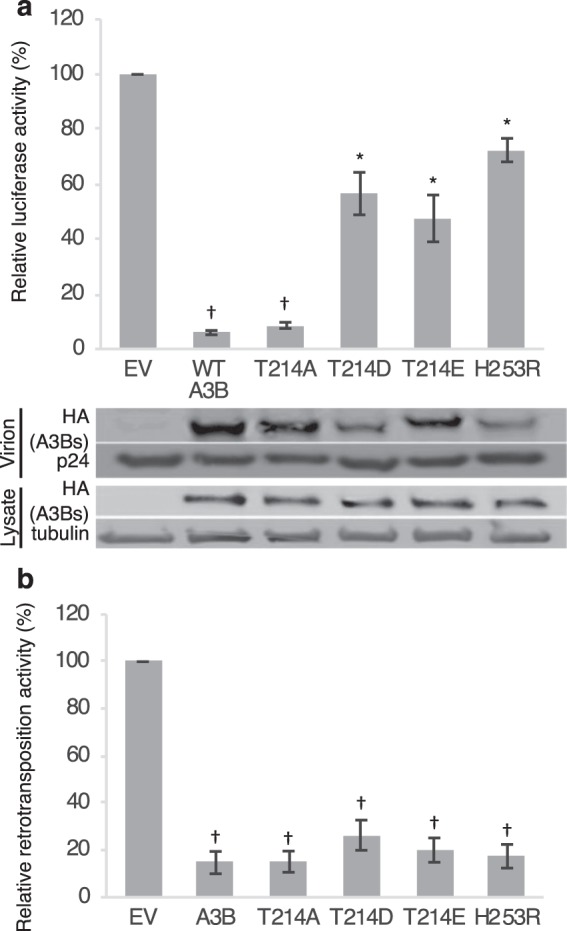
Phosphomimetic A3B mutants retain anti-retroviral activity and retrotransposition restriction. (**a**) VSV-G pseudotyped NL4-3 viruses were prepared by co-transfection of pNL4-3/Δenv/luc and VSV-G together with expression vectors for A3B or its mutants. HEK293T cells were infected with the produced viruses. Luciferase activity of the target cell lysate was measured and presented as ratio of infectivity reported to the EV (upper panel). Protein levels of A3B and mutants within the lysates or virions are comparable (lower panel) (**b**) A3B suppresses retrotransposition. Both WT and phosphomimetic mutants, and even the catalytically inactive H253R mutant, inhibit retrotransposition. Values represent the average of three independent experiments, bars indicate SE, and daggers (†) and asterisks (*) show statistically significant difference (p < 0.01 and p < 0.05, respectively).

Overexpressed A3B inhibits retroviral infectivity^2^. Originally, A3B and other APOBEC3s were thought to inhibit viral infection by inducing mutations in the viral genome via their CDA, but catalytically inactive A3B mutants nevertheless retain significant HIV-1 inhibitory activity^8^. Therefore, as phosphomimetic A3B mutants also lack CDA activity in cells and *in vitro*, we speculated that they also retain their retroviral inhibitory activity. We performed luciferase-based retroviral infection assays^36^. We infected HEK293T cells with reporter viruses containing WT A3B or mutants and measured their infectivity as luciferase activity in the cell lysate. WT A3B and the T214A mutant completely blocked HIV infectivity, whereas phosphomimetic mutants reduced it by approximately 50% (Fig. 5a). These data indicate that phosphomimetic A3B mutants partially retain their anti-retroviral activity.

### Phosphomimetic A3B mutants retain LINE1 retrotransposition restriction

A3B inhibits LINE1 (L1) retrotransposition^50^ even in the absence of its CDA activity^51^. To assess whether the phosphomimetic A3Bs inhibit L1 retrotransposition as well, we performed GFP-based L1 retrotransposition assays (Fig. 5b). In this system, L1 transcription, splicing, reverse transcription, and integration are necessary for EGFP expression, and retrotransposition can be monitored as percentage of EGFP positive cells^52^. EGPF-positivity of HEK293T cells transfected with expression vectors for WT A3B or mutants together with the EGFP-based L1 reporter construct, was analyzed by flow cytometry after 8 days in culture. We found that L1 retrotransposition occurred in about 5% of empty vector-transduced cells. WT A3B reduced retrotransposition 5- to 10-fold; the T214D, T214E, T214A and H253R mutants also restrict retrotransposition to similar extents as WT A3B. These data affirm that phosphomimetic A3B mutants retain their full anti-retrotransposition activity.

## Discussion

In this study, we demonstrate that PKA specifically phosphorylates A3B Thr214 and that PKA-mediated phosphorylation of A3B directly suppresses its CDA activity *in vitro* and *in vivo*. We also demonstrate that the phosphate group on Thr214 forces out cytosine in the substrate ssDNA by electrostatic repulsion. Finally, we prove that phosphomimetic A3B mutants lose their mutagenic activity in cells, while retaining their inhibitory activity against viral infection and retrotransposition.

We demonstrated that PKA-mediated phosphorylation of Thr214 suppresses its CDA activity *in vitro*. The initial FRET-based CDA assays were performed using cell lysates with overexpressed WT or mutant A3Bs, and they showed that phosphomimetic A3B mutants have almost no CDA activity. But this did not exclude the possibility of other mechanisms inhibiting the *in vitro* CDA reaction, such as cellular inhibitory factors specifically induced by the phosphomimetic A3Bs. We performed FRET-based and gel-based CDA assays using purified A3B-CTD WT or mutants, and confirmed that phosphomimetic A3B mutants indeed lost their CDA activities. We also quantified the CDA activity of *in vitro* phosphorylated A3B and found that PKA-induced phosphorylation at least partially reduced it. We surmise that our *in vitro* phosphorylation assays could not phosphorylate all A3B proteins, which is why we observed only a partial reduction of CDA activity in these assays.

To gain deeper insight on how PKA-mediated phosphorylation of A3B Thr214 suppresses its CDA activity, we performed molecular dynamics simulations (MDS) by computer-generated structural models of A3B WT and its mutants to visualize the ssDNA-A3B interaction and determine how it is affected by phosphorylation or mutation of Thr214. According to our data, the additional phosphate group hinders ssDNA entry within the catalytic pocket via electrostatic repulsion. The phosphomimetic mutants, T214D and T214E, lead to similar outcomes, suggesting that Thr214 phosphorylation inhibits A3B CDA activity by preventing ssDNA docking into its catalytic pocket. The conformational changes displayed by T214A could be explained by the lack of hydrogen bond formation between the amino acid and cytosine and support the typical functional alterations of this mutant.

At last, we analyzed whether PKA-mediated phosphorylation of A3B alters its biological functions. To begin with, through EGFP-based foreign DNA editing assays and then sequencing, we showed that phosphomimetic A3B mutants lose their mutagenic activity, proving that PKA-mediated phosphorylation of A3B also inhibits its CDA activity *in vivo*.

In contrast, phosphomimetic A3Bs have reduced, but still significant inhibitory activity against retroviral infection and an anti-retrotransposition activity comparable to that of WT A3B. A3G is a well-known potent retroviral infection inhibitory factor and its CDA activity is important for anti-retroviral activity, but catalytically inactive A3G also retains a degree of anti-retroviral activity^8,53–60^ based on reverse transcription suppression. Like with A3G, A3B CDA activity contributes to, but is not the only mechanism in HIV-1 restriction^3^. These reports support our data that catalytically inactive phosphomimetic A3B mutants still possess inhibitory activity against viral infection. As is the case with the anti-retroviral activity of A3B, Stenglein *et al*. reported that A3B CDA activity is not required to inhibit retrotransposition^51^ and our data regarding the A3B phosphomimetic mutants is consistent with this report. Overall, we show that Thr214 phosphorylated A3B loses its mutagenic activity, while it retains its anti-viral and anti-retrotransposition activity *in vivo*.

Several recent reports identified A3B as an intrinsic mutagen, and mutations induced by A3B are supposed to drive clonal evolution and malignant transformation of several types of cancers^10,15,21^. Therefore, to inhibit A3B CDA activity could be an attractive therapeutic strategy to counter A3B-mediated mutagenesis. On the other hand, A3B also acts as a potent inhibitory factor against viral infections and retrotranspositions. Furthermore, recent studies also reported that transposable elements including LINE-1 or the act of transposition itself were responsible for mutations in tumorigenesis^61,62^. Thus, PKA-mediated phosphorylation might be a better option to suppress A3B CDA activity rather than reducing the A3B expression by SIV-Vif or inhibiting the NF-κB pathway^24,25^.

In conclusion, our findings constitute the first evidence that the cytidine deaminase activity of A3B is potentially regulated by PKA-mediated phosphorylation at Thr214. In addition, the phosphomimetic A3B mutants retain its anti-viral and anti-retrotransposition functions *in vivo*. Further studies are required to investigate whether phosphorylation of A3B suppresses clonal evolution in particular cancers, including breast cancer and myeloma, and whether it improves the outcomes in these cancers.

## Experimental Procedures

### DNA constructs and cell cultures

pDON-EGFP and pCAG-GS AID, A3G, A3B, A3B H253R and H66/253R mutants have been described previously^20^. The expression vector for Uracil-DNA glycosylase inhibitor, pEF-UGI, was kindly provided by Dr. Reuben S Harris. A3B S46A, T214A, T214D and T214E were generated using KOD Fx Neo with primer pairs (S46A: 5′-GAAAATAAAGAGGGGCCGCGCCAATCTCCTTTGGGAC-3′ and 5′-GTCCCAAAGGAGATTGGCGCGGCCCCTCTTTATTTTC-3′; T214A: 5′-CTTCGACGGCGCCAGGCCTACTTGTGCTATGAG-3′ and 5′-CTCATAGCACAAGTAGGCCTGGCGCCGTCGAAG-3′; T214D: 5′-CTTCGACGGCGCCAGGACTACTTGTGCTATGAG-3′ and 5′-CTCATAGCACAAGTAGTCCTGGCGCCGTCGAAG-3′; T214E: 5′-CTTCGACGGCGCCAGGAGTACTTGTGCTATGAG-3′ and 5′-CTCATAGCACAAGTACTCCTGGCGCCGTCGAAG-3′). The PKACA fragment was PCR amplified from HEK293T cDNA using KOD Fx Neo with one pair of primers (5′-NNNNGCATGCATGGGCAACGCCGCCGCCGC-3′ and 5′-NNNNGGATCCCTAAAACTCAGAAAACTCCTTGCC-3′), and cloned into pcDNA3.1 with Nhe1/BamH1. PKACA K72H was generated using KOD Fx Neo with one pair of primers (5′-CCGGGAACCACTATGCCATGCACATCCTCGACAAACAGAAGGTGG-3′ and 5′-CCACCTTCTGTTTGTCGAGGATGTGCATGGCATAGTGGTTCCCGG-3′). HEK293T, HEK293T-eGFP and HeLa cells were maintained with Dulbecco’s Modified Eagle’s Medium (Nacalai tesque) containing 10% fetal bovine serum (FBS) and 1% penicillin, streptomycin, and glutamine (PSG).

### Co-immunoprecipitation assays

HEK293T cells were transfected with expression vectors for A3B-HA or its mutants with or without expression vectors for FLAG-PKACA using the XtremeGENE HP DNA Transfection Reagent (Roche). At 36 to 48 hours after transfection, cells were lysed with radioimmunoprecipitation (RIPA) buffer (50 mM Tris-HCl pH 8.0, 150 mM NaCl, 1% (v/v) Triton X-100, 0.1% (v/v) SDS, 0.1% (v/v) sodium deoxycholate, complete Protease Inhibitor Cocktail (Roche), PhosSTOP (Roche)) and immunoprecipitated using the Anti-HA (12CA5) antibody (Roche) or the anti-FLAG (M2) antibody (F3165 Sigma-Aldrich) along with SureBeads Protein A Magnetic Beads (Bio-Rad) at 4 °C, followed by immunoblotting with anti-HA (12CA5) Ab or anti-FLAG (M2) antibodies.

### *in vivo* phosphorylation assays

HEK293T cells were transfected with expression vectors for A3B-HA, or its mutants (S46A, or T214A) with or without FLAG-PKACA, or K72H PKACA, using the XtremeGENE HP DNA Transfection Reagent. At 36 to 48 hours post transfection, cells were lysed with GST lysis buffer (25 mM HEPES-NaOH pH 7.4, 150 mM NaCl, 0.5% (v/v) Triton X-100, 1 mM EDTA, 1 mM MgCl_2_, 1 mM ZnCl_2_, 10% (v/v) Glycerol, complete Protease Inhibitor Cocktail, PhosSTOP) and immunoprecipitated using the Anti-HA (12CA5) antibody (Roche) and Protein G Sepharose 4 Fast Flow at 4 °C, and subsequently analyzed by immunoblotting with anti phospho-PKA substrate rabbit Ab (anti-RXXS/T-p, #9611 Cell Signaling Technology,), or Anti-HA (12CA5) antibody (Roche).

### *in vitro* phosphorylation assays

Purified A3B-CTD and its mutants (T214A, T214D, T214E, and H253R) were produced using the wheat germ cell-free expression system^63,64^ and were provided by Cell Free Sciences (Supplementary Fig. 4). We incubated 400 ng of A3B-CTD or its mutants along with 200 ng of human recombinant PKACA (C8482 Sigma-Aldrich) in an appropriate buffer (20 mM Tris-HCl pH 7.5, 10 mM MgCl2, 100 ng/μl BSA, 1 mM DTT, 1 mM ATP, 1 mM cAMP) for 30 minutes at 30 °C, followed by immunoblotting with anti Phospho-PKA substrate rabbit Ab and anti-A3B rabbit Ab^24^.

### FRET-based cytidine deaminase assays

We transfected HEK293T cells with expression vectors for A3B or its mutants (T214A, T214D, T214E, or H253R) using XtremeGENE HP DNA Transfection Reagent. At 36 to 48 hours after transfection, cells were lysed with 200 μl of GST lysis buffer/well (6 well plate). 20 μl of each cell lysate and 1 pM of single-stranded DNA oligonucleotide (AAATTCAAATAGATAATGTGA) with 5′-attached FAM and 3′-attached TAMRA, were incubated with 0.02 units of Uracil DNA Glycosylase (UDG) (NEB) and 15 μl of reaction buffer (Tris-HCl pH 7.4, 10 mM EDTA) in Nunc 96-well black plates, for 2 hours at 37 °C. In this reaction, cytosine was deaminated to uracil, which was excised by UDG, creating an abasic site. This abasic site was hydrolytically cleaved in 100 mM NaOH for 30 minutes at 37 °C. We then added 3 μl of 4 M HCl and 37 μl of 2 M Tris-HCl pH 7.9 per well, and measured the fluorescence intensity using a plate reader (ARVO, Perkin-Elmer) with excitation at 485 nm and emission at 535 nm. For the FRET-based CDA assays using purified proteins, we used purified WT or mutant A3B instead of lysate, in the indicated amounts. Data shown in Fig. 2b,d are obtained from three independent experiments, and in Fig. 2f from two independent experiments. p values were calculated by T test, and p < 0.05 was defined as significant difference.

### Gel-based CDA assays

5 μl of lysate with overexpressing A3B WT or mutants and 1 pM of single strand DNA oligonucleotide (ATTATTATTATTCAAATGGATTTATTTATTTATTTATTTATTT) with 5′-attached FAM were incubated with 0.005 units of UDG and 3.75 μl of reaction buffer in 10 μl reaction volume for 2 hours at 37 °C. Subsequently, the oligo products were incubated in 100 mM NaOH for 30 minutes at 37 °C, stained with loading dye, and denatured, followed by electrophoresis in 20% Tris/urea-acrylamide gel, which is then visualized with the Gel Doc EZ Gel Documentation System (Bio-Rad).

### Immunohistochemical analysis

HeLa cells are transfected with expression vectors for WT A3B or its mutants and fixed in 4% paraformaldehyde containing PBS. Fixed cells were incubated with anti FLAG M2 antibody (Sigma-Aldrich F3165) for 1 hour, following anti-mouse IgG-Alexa Fluor 594 antibody (Abcam, ab150120) as secondary antibody for 1 hour and 4′,6-diamidino-2-phenylindole (DAPI). Cells are observed with a fluorescence microscope (BZ-9000, KEYENCE).

### Foreign DNA editing assays

HEK293T cells were transfected with pDON-EGFP, pEF-UGI, and expression vectors for A3B WT or its mutants, using the XtremeGENE HP DNA Transfection Reagent. After two-day cultures, total DNA was extracted using the Quick Gene DNA whole blood kit (KURABO). First round PCR was performed using the primers and rTaq DNA polymerase (Takara), with the following reaction profile: 30 s at 94 °C, 25 cycles of 30 s at 94 °C, 40 s at 62 °C, and 90 s at 72 °C, followed by 10 min at 72 °C^20^. The amplicons were separated by electrophoresis in 1% (w/v) agarose gel, and extracted with the FastGene Gel/PCR Extraction kit (NIPPON Genetics). We used 100 ng of first-round PCR products as templates for nested PCR using KOD Fx Neo (TOYOBO), with the following reaction profile: 5 min at 95 °C, 30 cycles of 10 s at 81–88 °C, 30 s at 62 °C, and 60 s at 72 °C, followed by 5 min at 72 °C. The amplicons derived at each of the indicated denaturation temperatures were then cloned into the pT7-blue vector (Novagen).

### Molecular dynamics simulations (MDS)

The initial structural model for constructing various monomeric A3Bs in an ssDNA-bound state was made by placing the ssDNA in the active center of the A3B mutant at a resolution of 1.72 Å (PDB number 5TD5)^46^ into that of the monomeric human A3B at a resolution of 1.73 Å (PDB number 5CQH)^45^ by using the tools in the Molecular Operating Environment (MOE) (Chemical Computing Group Inc., Montreal, Quebec, Canada). A3B structures with a phosphorylated residue or various single amino acid mutations were constructed with the MOE. MDS were done as described previously^65–67^. Briefly, the simulations were performed by the pmemd.cuda module in the Amber 16 program package^68^ with the amber ff14SB force field for the protein portion^69^, OL15 force field for the ssDNA^70^, and the TIP3P water model for simulations of aqueous solutions^71^. The AMBER/DYANA/MOLMOL phosphorylated amino acid library set^72^ was used for the force field of the phosphorylated threonine residue. Bond lengths involving hydrogen were constrained with SHAKE, an algorithm to satisfy for Newtonian motion^73^, and the time step for all MDS was set to 2 fs. A non-bonded cutoff of 10 Å was used. After calculations for heating up to 310 K for 20 ps using the NVT ensemble for constant volume, temperature, and number of particles in the system, simulations were executed using the NPT ensemble for constant pressure, temperature, and number of particles in the system at 1 barr, 310 K, and in 150 mM NaCl for 100 ns.

### Luciferase-based retrovirus infection assays

We prepared Luciferase reporter viruses lacking Vif by co-transfecting HEK293T cells with pNL43/ΔEnv/ΔVif-Luc plus pVSV-G, together with expression vectors for WT A3B, or its mutants. The supernatant was collected at 48 hours and the virus titer measured by p24 ELISA (RETROTEK, ZeptoMetrix). HEK293T cells were incubated for 24 hours with adjusted amounts of each virus, then lysed in passive lysis buffer (Promega) and checked for luciferase activity (ARVO, Perkin-Elmer). Values are the average of three independent experiments, and represented as percentage of the infectivity of the empty vector. p values were calculated by T test, and p < 0.05 was defined as significant difference.

### EGFP-based L1 retrotransposition assays

We transfected HEK293T cells with 0.1 μg of the respective A3B expression plasmids, 0.5 μg of the EGFP-based L1 reporter vector EF06R, and 0.4 μg of the empty vector. Transfected cells were maintained in puromycin (0.5 mg/ml) for 8 days. EGFP expression resulting from retrotransposition was analyzed by flow cytometry. Values are the average of three independent experiments, and represented as percentage of the retrotransposition observed in the empty vector sample. p values were calculated by T test, and p < 0.05 was defined as significant difference.

## Supplementary information

Supplementary Figures
