# Supplementary material for: Protein kinase A inhibits tumor mutator APOBEC3B through phosphorylation
Source: Sci Rep. 2019 Jun 5;9:8307. doi: 10.1038/s41598-019-44407-9 (PMC6549188; doi:10.1038/s41598-019-44407-9)
Supplement: Supplementary file 1 — Supplementary Figures [file 41598_2019_44407_MOESM1_ESM.pdf]

Supplementary Information for

Protein kinase A inhibits tumor mutator APOBEC3B through phosphorylation

Tadahiko Matsumoto[1], Kotaro Shirakawa[1], Masaru Yokoyama[2], Hirofumi Fukuda[1], Anamaria Daniela Sarca[1], Sukenao Koyabu[1], Hiroyuki Yamazaki[1], Yasuhiro Kazuma[1], Hiroyuki Matsui[1], Wataru Maruyama[1], Kayoko Nagata[1], Fumiko Tanabe[3], Masayuki Kobayashi[1], Keisuke Shindo[1], Ryo Morishita[3], Hironori Sato[2], Akifumi Takaori-Kondo[1]*

From the [1]Department of Hematology and Oncology, Graduate School of Medicine, Kyoto University, Kyoto 606-8507, Japan, [2]Laboratory of Viral Genomics, Pathogen Genomics Center, National Institute of Infectious Diseases, Tokyo 208-0011, Japan, [3]CellFree Sciences Co., Ltd, Ehime 790-8577, Japan.

AID	A	K	G	R	R	E	T_{27}	Y	-	L	C	Y	V
A3G	I	L	S	R	R	E	T_{32}	V	W	L	C	Y	E
A3G	V	R	G	R	H	E	T_{218}	Y	-	L	C	Y	E
A3B	K	I	K	R	G	R	S_{46}	N	-	L	L	W	D
A3B	V	L	R	R	R	Q	T_{214}	Y	-	L	C	Y	V

Figure S1. An alignment of consensus PKA phosphorylation motifs, R-X-X-T, found at AID T27, A3G T32, A3G T218, A3B S46 and A3B T214.

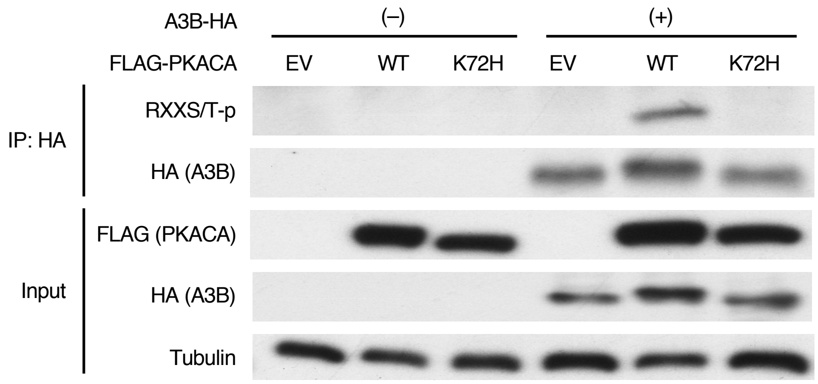

Figure S2. Kinase activity of PKACA is essential for PKA-induced phosphorylation of A3B. We carried out in vivo phosphorylation assays using WT and kinase-dead mutant PKACA,K72H. WT PKACA normally phosphorylates A3B, but K72H mutant doesn’t.

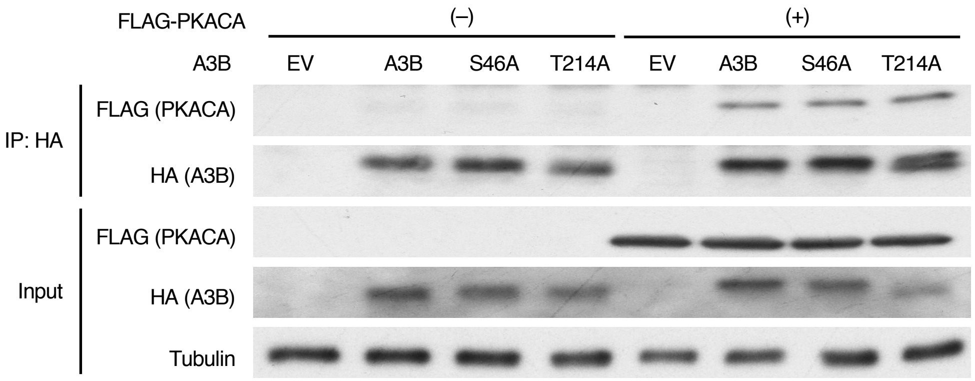

Figure S3. WT A3B, S46A, and T214A mutants are comparably bind to PKACA in cells. We performed co-immunoprecipitation assays with anti-HA antibody using lysate with overexpressed C-terminal HA tagged A3B or its mutant with or without N-terminal FLAG-tagged PKACA. The results showed that T214A non-phosphorylation mutant, as well as WT and S46A, almost equally bind to PKACA.

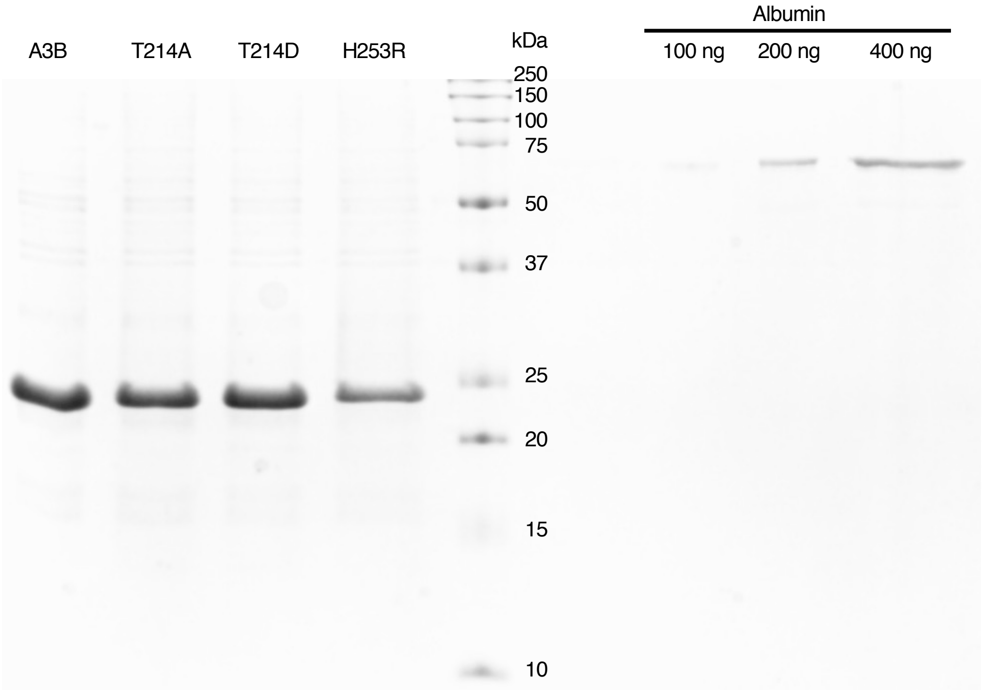

Figure S4. Coomassie-stained gel illustrating the purity of A3B enzymes used in Figures 1 and 2. SDS-PAGE, followed by Coomassie staining, was performed using purified C-terminal domain of A3B.

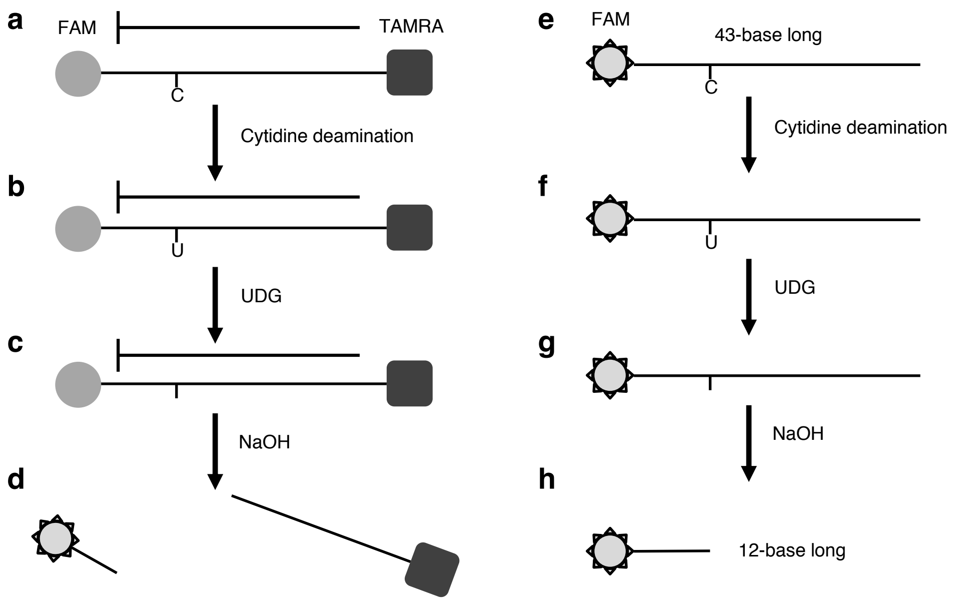

Figure S5. Overview of *in vitro* CDA assays. (Left column, a-d) Overview of the FRET-based CDA assay. (a) The substrate oligo nucleotide contains one CDA target, cytosine, and two fluorescent groups attached, 5'-FAM and 3'-TAMRA. In this form, TAMRA quenches the fluorescence of FAM. (b) Deamination and (c) uracil excision generate an abasic site. (d) NaOH hydrolytically breaks the DNA backbone, and FAM escapes from TAMRA quenching. **(Right column, e-h) Overview of the gel-based CDA assay.** The reaction is the same as in the left column. The substrate oligo is 43-base long, and has only one fluorescence group, FAM, at its 5' end. After deamination, uracil excision, and hydrolytic break, the product oligo is 12-base long. These oligos are analyzed in 20% Tris/urea acrylamide gel electrophoresis.

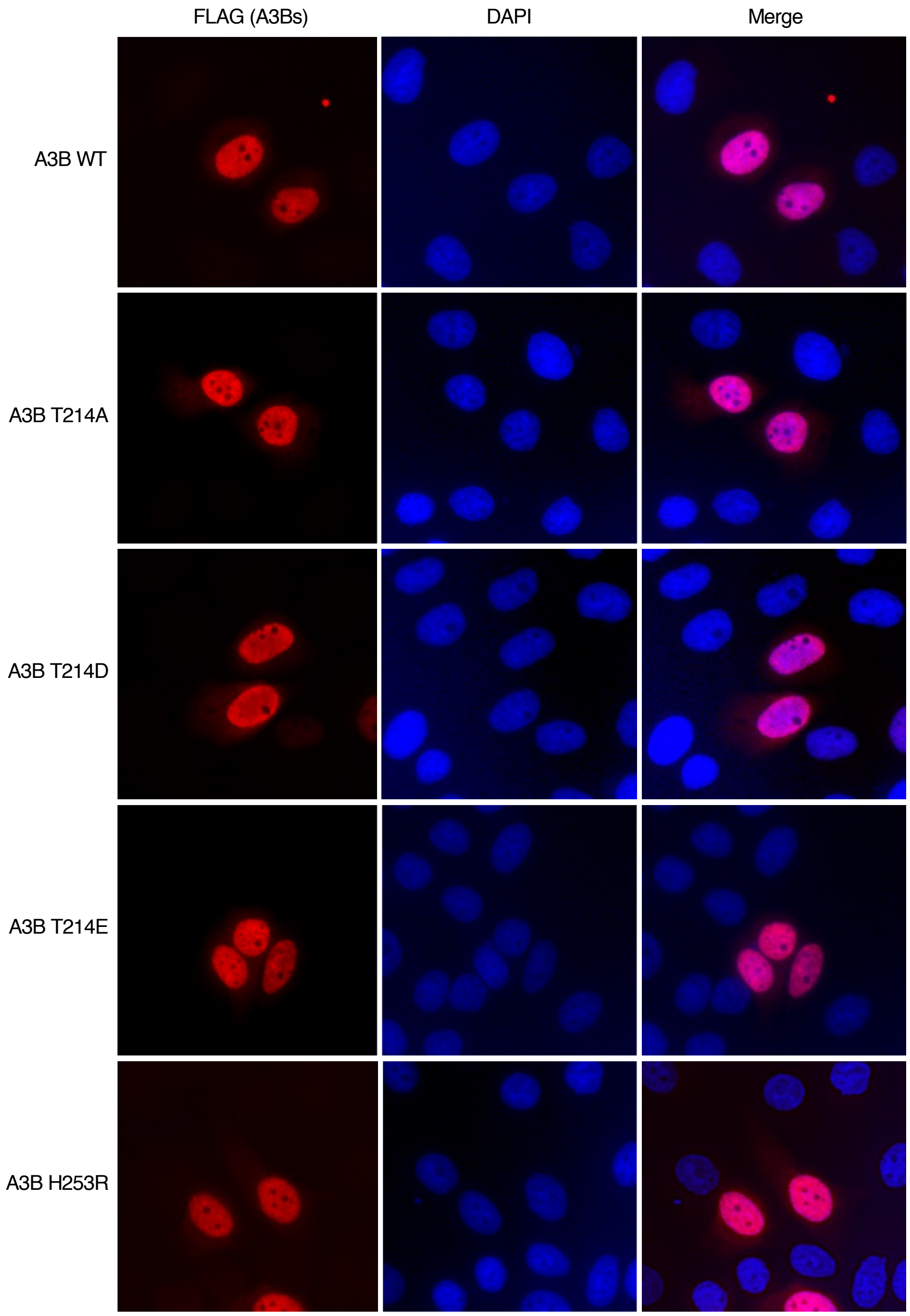

Figure S6. A3B WT and mutants localize in the nucleus in HeLa cells. HeLa cells were transfected with expression vectors for WT or mutant C-terminal FLAG-tagged A3B, as indicated. Immunofluorescent A3B staining was performed using the anti-FLAG antibody (M2) and secondary anti-mouse IgG-Alexa fluor 594 antibody, and DAPI for the nucleus.
